# Acute kidney injury in neurocritical care

**DOI:** 10.1186/s13054-023-04632-1

**Published:** 2023-09-03

**Authors:** Faeq Husain-Syed, Tomonori Takeuchi, Javier A. Neyra, Gonzalo Ramírez-Guerrero, Mitchell H. Rosner, Claudio Ronco, Ashita J. Tolwani

**Affiliations:** 1 Division of Nephrology, University of Virginia School of Medicine, 1300 Jefferson Park Avenue, Charlottesville, VA 22908 USA; 2 Department of Internal Medicine II, University Hospital Giessen and Marburg, Justus-Liebig-University Giessen, Klinikstrasse 33, 35392 Giessen, Germany; 3 Division of Nephrology, University of Alabama at Birmingham, 1720 2nd Avenue South, Birmingham, AL 35294 USA; 4 Department of Health Policy and Informatics, Tokyo Medical and Dental University, 1-5-45 Yushima, Bunkyo Ku, Tokyo, 113-8510 Japan; 5 Critical Care Unit, Carlos Van Buren Hospital, San Ignacio 725, Valparaíso, Chile; 6 Dialysis and Renal Transplant Unit, Carlos Van Buren Hospital, San Ignacio 725, Valparaíso, Chile; 7 Department of Medicine, Universidad de Valparaíso, Hontaneda 2653, Valparaíso, Chile; 8 Department of Medicine (DIMED), Università di Padova, Via Giustiniani, 2, 35128 Padua, Italy; 9 International Renal Research Institute of Vicenza, Department of Nephrology, Dialysis and Transplantation, San Bortolo Hospital, Via Rodolfi, 37, 36100 Vicenza, Italy

**Keywords:** Dialysis disequilibrium syndrome, Intracerebral hemorrhage, Renal replacement therapy, Stroke, Subarachnoid hemorrhage, Traumatic brain injury, Uremia

## Abstract

Approximately 20% of patients with acute brain injury (ABI) also experience acute kidney injury (AKI), which worsens their outcomes. The metabolic and inflammatory changes associated with AKI likely contribute to prolonged brain injury and edema. As a result, recognizing its presence is important for effectively managing ABI and its sequelae. This review discusses the occurrence and effects of AKI in critically ill adults with neurological conditions, outlines potential mechanisms connecting AKI and ABI progression, and highlights AKI management principles. Tailored approaches include optimizing blood pressure, managing intracranial pressure, adjusting medication dosages, and assessing the type of administered fluids. Preventive measures include avoiding nephrotoxic drugs, improving hemodynamic and fluid balance, and addressing coexisting AKI syndromes. ABI patients undergoing renal replacement therapy (RRT) are more susceptible to neurological complications. RRT can negatively impact cerebral blood flow, intracranial pressure, and brain tissue oxygenation, with effects tied to specific RRT methods. Continuous RRT is favored for better hemodynamic stability and lower risk of dialysis disequilibrium syndrome. Potential RRT modifications for ABI patients include adjusted dialysate and blood flow rates, osmotherapy, and alternate anticoagulation methods. Future research should explore whether these strategies enhance outcomes and if using novel AKI biomarkers can mitigate AKI-related complications in ABI patients.

**Graphical abstract:**

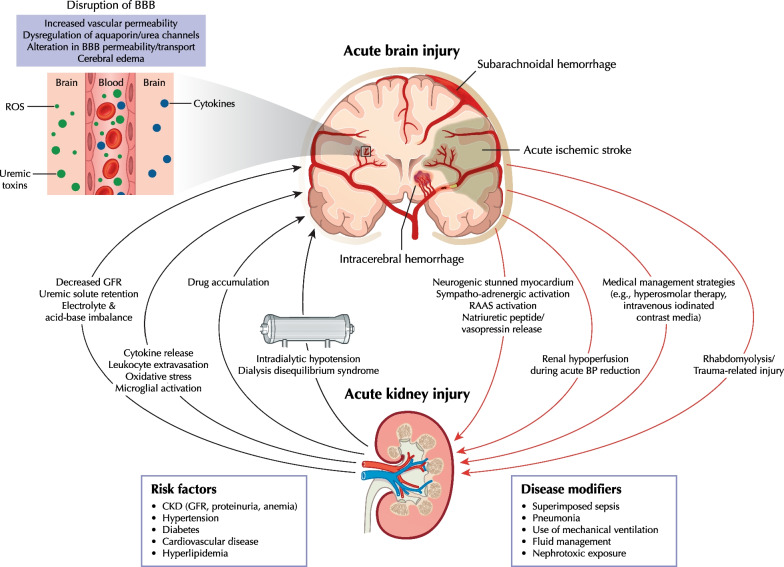

**Supplementary Information:**

The online version contains supplementary material available at 10.1186/s13054-023-04632-1.

## Background

Acute kidney injury (AKI) in intensive care units (ICUs) is an independent risk factor for death. Reported mortality rates from AKI with renal replacement therapy (RRT; 40–55%) are higher than the mortality rates due to ICU-related myocardial infarction (20%), sepsis without AKI (15–25%), or acute respiratory distress syndrome requiring mechanical ventilation (30–40%) [1]. This high mortality rate is attributed to the systemic impact of AKI on the brain, heart, lungs, liver, and gastrointestinal tract, linking AKI to various syndromes (Additional file 1) [2]. Beyond the acute phase, AKI increases the likelihood of chronic kidney disease (CKD), cardiovascular complications, recurrent AKI, and functional impairment [3].

In the context of neurocritical care, AKI diagnosis holds significance for acute brain injury (ABI) management. This article summarizes the epidemiology and outcomes of AKI in neurocritically ill adults admitted to the ICU, focusing on prevalent ABI conditions like traumatic brain injury (TBI), aneurysmal subarachnoid hemorrhage (SAH), intracerebral hemorrhage (ICH), and acute stroke. The review explores potential AKI mechanisms and their involvement in reciprocal ABI progression, while also highlighting essential principles in AKI management. Additionally, considerations for managing RRT in the context of ABI are discussed. Our literature search strategy is provided in Additional file 2.

## AKI epidemiology and outcomes in neurocritical care

Studies on AKI prevalence in neurocritical care are few, and their results vary with AKI definition [4–6] (Additional file 3). Current AKI diagnosis and classification are based on the 2012 KDIGO (*Kidney Disease: Improving Global Outcomes*) consensus criteria [6] (Additional file 4; left panel). This three-stage severity classification system is based on changes in serum creatinine (SCr) concentration and urine output (UOP). However, most neurocritical care studies only used SCr-based definitions because SCr data are readily available in electronic health records, missing a substantial proportion of patients with AKI [7].

In a multinational study spanning 97 ICUs and encompassing more than 1800 patients, with 25.9% primarily diagnosed with neurological symptoms, 57% of the entire cohort developed AKI by KDIGO criteria within the initial week of admission [8]. Among these, 39% experienced stages 2–3 AKI, and 13.5% required RRT, accounting for 23.5% of AKI cases [8]. While insights into AKI incidence among neurocritical patients mainly stem from retrospective studies in single centers and exclude cases diagnosed with AKI upon admission, a recent study in a mixed neurological/neurosurgical ICU reported an overall AKI occurrence of 23.5% utilizing the SCr/UOP KDIGO criteria [9]. Interestingly, similar AKI rates were noted among specific brain pathologies, including TBI, 11.9–26.7% (KDIGO, SCr only) [10–14] and 17.7% (SCr/UOP KDIGO) [15]; acute stroke, 20.9–43.3% (KDIGO, SCr only) [16, 17]; non-traumatic aneurysmal SAH, 16.7–23.7% (KDIGO, SCr only) [18, 19], and ICH, 20.0–29.9% [18, 20–22]. The few studies assessing RRT in patients with ABI reported overall rates of 0.3–5.6% among neurocritical patients [17, 23]. Irrespective of the criteria employed and the specific brain condition under consideration, AKI severity is associated with increased mortality, obstacles to rehabilitation care, increased likelihood of disability at discharge, and potential downstream effects on healthcare services [8, 10, 24–27].

Data on renal recovery after AKI post-ABI are scarce [18], and long-term renal outcomes after AKI in ABI patients remain unknown.

## AKI causes and pathophysiological mechanisms

Clinicians must consider various AKI causes specific to both neurocritical and non-neurological ICU patients (Additional file 1). The Graphical Abstract summarizes key brain-kidney interaction pathways in neurocritical care that could lead to reciprocal ABI and AKI progression.

CKD plays a significant role as a risk factor for AKI in neurocritical patients, with those having CKD displaying a fivefold higher prevalence of cerebrovascular disease compared to those without CKD [10, 26, 28]. These data are unsurprising, given that the kidneys and brain share common vascular risk factors, including hypertension, diabetes, and hyperlipidemia. Notably, proteinuria of ≥ 30 mg/dL and CKD stages 3–4 are established risk factors for cerebrovascular disease, which, in turn, increases the likelihood of AKI [29, 30]. A study on patients with spontaneous ICH found that individuals with CKD experience worse functional outcomes (OR 1.91; 95% CI 1.04–3.52) and higher mortality (OR 3.33; 95% CI 1.76–6.27) at 12 months post-ICH compared to those without CKD [31]. While TBI patients tend to be younger and possess normal pre-existing renal function, patients with acute stroke, aneurysmal SAH, and ICH are generally older and more likely to have underlying CKD.

ABI can be complicated by neurogenic stunned myocardium [32], which is a transient and diffuse form of left ventricular cardiomyopathy, likely triggered by autonomic nervous system activation with excess catecholamine release, impaired myocardial glucose metabolism secondary to neurocardiogenic injury, and coronary microvascular dysfunction [33, 34]. Patients with this condition exhibit a wide range of cardiac abnormalities including arrhythmias and ventricular dysfunction [33, 34], increasing the risk of AKI due to reduced cardiac output, pulmonary edema, and prolonged vasopressor support [35]. However, the prevalence of AKI in such patients has not been described. Autonomic nervous system imbalance also contributes to sympatho-adrenergic drive and renin–angiotensin–aldosterone system activation and vasopressin release, resulting in renal vasoconstriction, enhanced sodium and water reabsorption, and decreased renal blood flow and glomerular filtration rate (GFR) [36]. Neurogenic stunned myocardium prevalence in patients with SAH is high and estimated at ~ 30% [32].

The 2020 Neurocritical Care Society (NCS) guidelines suggest, with low evidence, using hyperosmolar therapy for initial management of cerebral edema in neurocritical patients with SAH, TBI, acute ischemic stroke, and ICH [37]. While mannitol is the most frequently administered hyperosmolar solution, its use, particularly at high doses, has been associated with an increased risk of AKI in such patients [38–40]. Risk factors for mannitol-associated AKI include higher illness severity, heart failure, diabetes, use of diuretics, and lower baseline GFR [38, 41]. The precise mechanism for mannitol-associated AKI is not well defined but may be related to increased serum osmolality [42] and appears to be dose-related [11]. The 2020 NCS guidelines recommend hypertonic sodium solution over mannitol as it might be more effective in reducing ICP or cerebral edema ABI [37]; however, its usage can lead to hypernatremia and hyperchloremia, both of which have been associated with an increased AKI risk [19, 43, 44]. However, the effects of high chloride content on the kidneys have been debated [45, 46]. While experimental studies suggest it causes renal vasoconstriction and decreased GFR [47–49], a post-hoc analyses of the EPO-TBI and COBI trials demonstrated no association between hypertonic sodium solution and AKI [11, 14]. Notably, randomized controlled studies specifically focusing on AKI and acid–base balance outcomes related to hyperosmolar therapy in neurocritical care are lacking.

Intravenous contrast might be a concern in patients with ABI due to increased AKI risk, particularly in patients with SAH who might receive substantial amounts of contrast due to repeated computed tomography angiography scans for the diagnosis and/or management of cerebral vasospasm/delayed cerebral ischemia. However, existing evidence suggests that the risks associated with low- or iso-osmolar intravenous contrast agents are minimal, and AKI is infrequent in patients with eGFR of ≥ 30 mL/min/1.73 m^2^ [50, 51]. Intraarterial contrast administration poses a greater risk for AKI because such procedures often require larger doses that reach the renal arteries at high concentrations and potentially lead to atheroembolic complications [52].

Sepsis and nosocomial infections, especially ventilator-associated pneumonia, are common in neurocritical patients. A retrospective single-center study reported sepsis (75%) and respiratory infections (68%) as the main non-neurological complications in patients with TBI [53]. Although data on patients with ABI are limited, infectious complications are considered the main AKI disease modifiers during ICU stay [16, 26], in line with general ICU populations [8].

Myoglobin-associated rhabdomyolysis and renal toxicity should be considered in patients with TBI. A recent multicenter registry study found rhabdomyolysis present in 3.8% of patients with TBI and independently associated with AKI occurrence [54]. Moreover, while rhabdomyolysis and elevated serum/urinary myoglobin correlate with AKI occurrence, they also correlate with trauma severity and other renal insults. Hence, the direct contribution of rhabdomyolysis to kidney injury is often uncertain. The causative role of myoglobin toxicity in AKI is more certain in cases with severe rhabdomyolysis (creatinine kinase > 15,000 IU/L), which rarely occurs in patients with TBI.

## Brain injury progression following AKI

Experimental evidence suggests that while AKI is an indicator of illness severity, it can cause further organ dysfunction and damage. Despite limitations due to interspecies differences, animal kidney injury models have been used extensively to elucidate the mechanisms leading to remote organ dysfunction after AKI (Table 1).

**Table 1:** Experimental studies on brain-kidney cross-talk

Study	Model	Method	Findings
Arieff et al. [55]	Dog	Bilateral urethral ligation; analyses 72 h later	Increased calcium content in the gray and white matter of the brain after AKI, which was prevented by TPTX; administration of parathyroid extract to normal and post-TPTX dogs associated with an increase in brain calcium; hemodialysis significantly reduced brain calcium content but values remained significantly above normal
Jeppsson et al. [56]	Rat	AKI model: unilateral nephrectomy and renal artery occlusion of the remaining kidney for 70 min; CKD model: unilateral nephrectomy and 70–80% devascularization of the remaining kidney; analyses 2 weeks later	Reduced plasma valine and threonine and increased brain phenylalanine, tyrosine, and histidine
Trachtman et al. [57]	Rat	Bilateral urethral ligation; analyses 8 h and 48 h later	Decreased brain water at 8 h and increased organic osmolyte in the brain at 48 h
Silver et al. [58]	Rat	Bilateral urethral ligation; analyses 42 h later	Increased brain water content in dialyzed over non-dialyzed AKI rats; no significant change in brain organic osmolytes
Adachi et al. [55]	Rat	Bilateral renal artery occlusion vs. sham-operation; analyses 48 h later	Decreased dopamine turnover in striatum, mesencephalon, and hypothalamus, which correlated with impaired motor activity; unchanged cerebral norepinephrine and serotonin turnover and brain water content
Liu et al. [59]	Mouse	Bilateral renal IRI for 60 min vs. sham-operation; analyses 24 h later	In mice with AKI: disrupted blood–brain barrier, increased neuronal pyknosis and microgliosis, increased keratinocyte-derived chemoattractant and G-CSF in the cerebral cortex and hippocampus, and elevated expression of glial fibrillary acidic protein in astrocytes in the cortex and corpus callosum
Palkovits et al. [60]	Rat	Bilateral renal IRI, bilateral urethral ligation, and drug-induced AKI vs. sham; analyses 24 h later	Moderate increase in neuronal activation in the biogenic amine expressing cell group
Salama et al. [61]	Rat	Bilateral renal IRI	Increased TLR-4 expression within the hippocampus and striatum
Chou et al. [62]	Mouse	Bilateral renal IRI for 60 min vs. sham-operation; analyses 2 h and 24 h later	In mice with AKI: higher serum and brain levels of KS, G-CSF, and MCP-1, increased brain vascular permeability, and altered genes expression in the hippocampus 2 h after reperfusion before changes in SCr
Arieff et al. [55]	Dog	Bilateral urethral ligation; analyses 72 h later	Increased calcium content in the gray and white matter of the brain after AKI, which was prevented by TPTX; administration of parathyroid extract to normal and post-TPTX dogs associated with an increase in brain calcium; hemodialysis significantly reduced brain calcium content but values remained significantly above normal
Jeppsson et al. [56]	Rat	AKI model: unilateral nephrectomy and renal artery occlusion of the remaining kidney for 70 min; CKD model: unilateral nephrectomy and 70%–80% devascularization of the remaining kidney; analyses 2 weeks later	Reduced plasma valine and threonine and increased brain phenylalanine, tyrosine, and histidine
Trachtman et al. [57]	Rat	Bilateral urethral ligation; analyses 8 h and 48 h later	Decreased brain water at 8 h and increased organic osmolyte in the brain at 48 h
Silver et al. [58]	Rat	Bilateral urethral ligation; analyses 42 h later	Increased brain water content in dialyzed over non-dialyzed AKI rats; no significant change in brain organic osmolytes
Adachi et al. [55]	Rat	Bilateral renal artery occlusion vs. sham-operation; analyses 48 h later	Decreased dopamine turnover in striatum, mesencephalon, and hypothalamus, which correlated with impaired motor activity; unchanged cerebral norepinephrine and serotonin turnover and brain water content
Liu et al. [59]	Mouse	Bilateral renal IRI for 60 min vs. sham-operation; analyses 24 h later	In mice with AKI: disrupted blood–brain barrier, increased neuronal pyknosis and microgliosis, increased keratinocyte-derived chemoattractant and G-CSF in the cerebral cortex and hippocampus, and elevated expression of glial fibrillary acidic protein in astrocytes in the cortex and corpus callosum
Palkovits et al. [60]	Rat	Bilateral renal IRI, bilateral urethral ligation, and drug-induced AKI vs. sham; analyses 24 h later	Moderate increase in neuronal activation in the biogenic amine expressing cell group
Salama et al. [61]	Rat	Bilateral renal IRI	Increased TLR-4 expression within the hippocampus and striatum
Chou et al. [62]	Mouse	Bilateral renal IRI for 60 min vs. sham-operation; analyses 2 h and 24 h later	In mice with AKI: higher serum and brain levels of KS, G-CSF, and MCP-1, increased brain vascular permeability, and altered genes expression in the hippocampus 2 h after reperfusion before changes in SCr

*AKI* acute kidney injury, *CKD* chronic kidney disease, *G-CSF* granulocyte-colony stimulating factor, *IRI* ischemia–reperfusion injury, *KS* keratinocyte-derived chemokine, *MCP-1* monocyte chemoattractant protein-1, *SCr* serum creatinine, *TLR-4* toll-like receptor-4, *TPTX* thyroparathyroidectomy

AKI causes systemic inflammation by generating proinflammatory cytokines and reducing cytokine clearance [63, 64]. These inflammatory processes contribute to AKI initiation and likely perpetuate and extend brain injury. Consequently, the brain and kidneys might interact during AKI by amplifying cytokine-induced damage and oxidative stress, extravasating leukocytes, and dysregulating cerebral aquaporin channels [65]. The various proinflammatory and metabolic changes observed in AKI (e.g., waste solute retention, disturbances of inorganic solute metabolism, and reduced drug clearance) can disrupt the blood–brain barrier. This disruption may lead to an influx of water and the accumulation of inflammatory cells, cytokines, and neurotoxic substances in the central nervous system. This accumulation can result in cerebral edema, inflammation, hemorrhage, and even death [66]. Animal models have supported this mechanism, demonstrating blood–brain barrier disruption [59], microvascular protein leakage, microglial cell activation [59], hippocampal injury, inflammation characterized by neuronal pyknosis [62], and increased soluble inflammatory proteins in the cerebral cortex and hippocampus [59] following renal ischemia–reperfusion injury-induced AKI. Importantly, neuronal pyknosis did not increase in corresponding animal models of acute liver injury, suggesting that some observed effects are relatively specific to AKI and not associated with generalized inflammation following acute organ injury [59]. Functionally, mice with AKI showed impaired locomotor function that correlated with renal ischemia duration [59]. Alterations in neurotransmitter secretion and uptake during AKI might worsen brain injury and dysfunction [66]. Furthermore, sepsis and liver failure, which are frequently observed in critical illness, can exacerbate brain injury and/or AKI and contribute to multiple organ dysfunction [67].

## AKI management

The underlying cause of AKI should be identified promptly, paying special attention to reversible causes. AKI-related syndromes (Additional file 1) pose a major challenge to AKI management, so regular re-evaluation is required for adaptive management. Since current evidence does not suggest that AKI in neurocritical patients should be managed differently from AKI in other critically ill populations, KDIGO-bundle recommendations (i.e., reduce nephrotoxic agents, monitor SCr/UOP, discontinue renin-angiotensin-system blockers, optimize fluid status) are considered appropriate [68]. No externally-validated scoring system is available to evaluate AKI risk in neurocritical patients.

### Hemodynamic management

Blood pressure (BP) targets should consider premorbid BP. The potential benefits of increased renal perfusion must be weighed against potentially deleterious effects on cerebral perfusion. Cerebral autoregulation is impaired in approximately one-third of patients with TBI [69], in whom a rise in mean arterial pressure (MAP) might increase the ICP due to hyperemia, while a drop in MAP might lead to cerebral hypoperfusion. Although targeting high MAP (≥ 80 mmHg) in patients with sepsis and chronic hypertension could benefit renal outcomes [70], careful evaluation of its effects on ICP and, consequently, on cerebral perfusion pressure (CPP) is required. As disease-specific data are lacking, CPP targets for ABI are usually derived from TBI guidelines, which recommend maintaining CPP at 60–70 mmHg and assessing cerebral autoregulation to individualized CPP targets [71, 72].

Norepinephrine is the first-line vasopressor used in sepsis with organ dysfunction and ABI [73, 74]. Data suggest that vasopressin could benefit some sepsis-associated AKI subtypes, but its role in patients with ABI and AKI is not fully known [75, 76]. Notably, vasopressin should be used cautiously as it might increase the risk of hyponatremia (and subsequent cerebral edema).

The magnitude of acute systolic blood pressure (SBP) reduction in patients with ICH, aimed at limiting hematoma growth, requires careful monitoring. According to the 2022 guidelines from the American Heart Association/American Stroke Association, reducing acute SBP to 140 mmHg and maintaining it within the range of 130–150 mmHg is regarded as safe and potentially beneficial for enhancing functional outcomes in patients with mild-to-moderate ICH who initially present with an SBP between 150–220 mmHg [77]. Because chronic hypertension shifts the plateau of the renal autoregulatory curve to higher levels [78], a sudden decrease in blood pressure (BP) could lead to significantly compromised tissue perfusion. A targeted stepwise BP reduction rather than absolute targeted value could optimize renal perfusion and mitigate AKI. In a post-hoc analysis of ATACH-II, which included patients with initial systolic blood pressure (SBP) of ≥ 220 mmHg (22.8% of the group), a significant reduction in SBP (110–139 mmHg) resulted in a higher rate of neurological dysfunction at 24 h and more renal complications by the seventh day of discharge [79]. This was compared to standard SBP lowering (140–179 mmHg), without any benefits in reducing hematoma growth at 24 h or rates of death or severe disability at 90 days [79]. This suggests that cautious lowering of blood pressure might be necessary for this specific subgroup. Subsequent analysis of ATACH-II indicated that a baseline SCr ≥ 1.25 mg/dL and higher intravenous nicardipine doses were associated with increased risk for AKI [80]. Accordingly, another study on patients with ICH indicated that an intensive SBP reduction to > 90 mmHg in the first 12 h increases the risk of AKI regardless of preexisting CKD [21].

### Fluid management

Much of the evidence on fluid management in ABI is derived from TBI guidelines. A negative fluid balance has been associated with adverse outcomes in patients with TBI [81], whereas fluid overload can cause systemic complications or cerebral edema and increased ICP [81, 82]. A multicenter study evaluating variable fluid management in patients with TBI found that incrementally positive fluid balance was associated with increased ICU mortality (OR, 1.10 per 0.1 L increase; 95% CI 1.07–1.12) and poor functional outcomes (OR, 1.04 per 0.1 L increase; 95% CI 1.02–1.05) [83]. Notably, these data likely represent confounding by indication, as sicker patients are more likely to receive additional volume. While the study did not assess renal outcomes, patients receiving a mean daily fluid balance of ≥ 0.37 L were more likely to undergo RRT than those with < 0.37 L (4% vs. 2%; *P* = 0.021) [83]. Reports on the association between fluid balance and renal outcomes in ABI are scarce. However, in non-neurological critically ill patients, the relationship between fluid overload and AKI is well-established due to factors like venous congestion, increased renal interstitial pressure, and decreased renal blood flow and GFR as observed in patients with cardiorenal syndrome [84].

The 2018 ESICM (*European Society of Intensive Care Medicine*) consensus statement recommends using MAP, fluid balance, and multimodal monitoring (e.g., ICP, brain tissue oxygen tension, autoregulatory status) to optimize fluid therapy in neurocritically ill patients [85]. Point-of-care focused ultrasonography is increasingly used to determine fluid status rapidly, facilitating personalized fluid management when appropriate [86]; however, their role in the neurocritical care setting has not been defined.

Several studies have focused on the administered fluid type. Current data do not conclusively support routine use of balanced crystalloid solutions over 0.9% saline to reduce the risk of AKI and RRT in critically ill patients. A recent meta-analysis showed that the risk ratios of developing AKI and of being treated with RRT with balanced crystalloids compared with 0.9% saline were 0.96 (95% CI 0.89–1.02) and 0.95 (95% CI 0.81–1.11), respectively [87]. However, the 2018 ESICM consensus statement recommends crystalloids as maintenance and resuscitation fluids in neurocritical care patients while not recommending albumin and hypotonic solutions [85]. Small single-center randomized trials in patients with SAH [88] and TBI [89] found that balanced crystalloids reduced the hyperchloremia rate compared to 0.9% saline. Synthetic colloids (e.g., starch, gelatin) should be avoided in patients with ABI as they increase the risk of AKI and death [85, 90].

### Hyperosmolar therapy

Hypernatremia and hyperchloremia are common complications in patients with ABI and risk factors for AKI and excess mortality [18, 19]. Consequently, fluid choice should be informed by the need to correct the specific electrolyte and acid–base imbalances. SCr and UOP should be closely monitored in patients receiving hyperosmolar therapy due to its strong association with AKI occurrence [6, 37]. The 2020 NCS guidelines recommend an upper serum sodium range of 150–155 mmol/L and chloride range of 110–115 mmol/L to decrease the risk of AKI In patients receiving hypertonic sodium solutions [37]. Bolus administration could be considered over continuous hypertonic sodium solution infusion as it could lead to fewer chloride values aberrations [20, 91]. An osmolality threshold of ≥ 320 mOsm/L has been suggested to increase the risk of AKI in patients receiving mannitol infusion [42]; however, this threshold has recently been questioned [41].

### Nephrotoxic exposure and drug dosing

Medications should be closely reviewed for potentially nephrotoxic agents, which should be discontinued or substituted with less nephrotoxic drugs. However, potentially nephrotoxic agents, e.g., intravenous contrast, should still be used in patients with ABI if the information gained could have important therapeutic implications. The 2020 American College of Radiology and National Kidney Foundation guidelines recommend prophylactic 0.9% saline before and after intravenous contrast exposure in patients with eGFR < 30 mL/min/1.73 m^2^ or recent AKI to reduce the risk of AKI [50]. Drug-induced acute interstitial nephritis (e.g., due to anticonvulsants or antibiotics) must also be considered [92, 93].

Augmented renal clearance of > 130 mL/min/1.73 m^2^ is common in patients with ABI. The 74% (95% CI 55–87) pooled prevalence of augmented renal clearance in neurocritical care patients reported in a recent meta-analysis is higher than in any other critical care population [94]. Postulated mechanisms that promote an augmented renal clearance in ABI patients include increased cardiac output, high serum atrial natriuretic peptides, and increased hypothalamus–pituitary–adrenal axis activity with elevated levels of cortisol and catecholamines [94, 95]. Augmented renal clearance has important implications for drug dosing in patients with ABI and may lead to underdosing of levetiracetam and vancomycin [96, 97]; therefore, clinicians should monitor renal-eliminated medications to achieve target trough concentrations.

### Biomarkers for AKI risk assessment

Factors contributing to AKI development and progression could be modified by incorporating novel biomarkers of early tubular stress or damage when clinical interventions or exposures increase the risk of AKI progression. Whether early identification of these patients could help reduce ABI-associated morbidity remains to be determined. A recent Acute Dialysis Quality Initiative consensus statement suggested that the AKI definition should be augmented by integrating novel AKI biomarkers into its risk-classification (Additional file 4; right panel) [98]. Although many candidate biomarkers exist (Additional file 5), prospective validation and implementation are needed. Among the few biomarkers studied in neurocritical care, cystatin C, neutrophil gelatinase-associated lipocalin, and liver-type fatty acid-binding protein at admission have been associated with increased risk of AKI in patients with TBI or stroke [99–102].

## Renal replacement therapy

Patients with ABI may require RRT to manage the consequences of impaired renal function, including electrolyte imbalances, metabolic acidosis, and fluid overload. However, the use of RRT can impact cerebral blood flow, CPP, ICP, and brain tissue oxygenation, potentially leading to neurological complications. The effect on these factors depends on the specific type of RRT and its outcomes, which calls for special consideration in ABI patients [103].

### Effects of RRT on the brain

RRT might lead to exacerbation of cerebral edema through the “reverse urea effect” or dialysis disequilibrium syndrome (DDS). During the initial stages of RRT, effective osmolytes such as urea are rapidly removed from the blood, creating an osmotic gradient between the blood and brain tissue. As brain cells have a relatively slow transport rate through cell membranes, this gradient causes water to move into the brain tissue, resulting in cerebral edema [104]. Additionally, elevated bicarbonate levels in dialysate and rapid rise in pH may induce paradoxical intracellular acidosis. This phenomenon occurs when bicarbonate-derived carbon dioxide crosses the cell membrane, leading to neuronal swelling and cerebral edema [105]. DDS and cerebral edema could lead to brain herniation or decreased CPP in patients with ABI.

Intradialytic hypotension, defined as a decrease in SBP by ≥ 20 mmHg or a reduction in MAP by ≥ 10 mmHg with associated symptoms [106], might occur during RRT and affect CPP because BP is its key determinant. It occurs when the body's hemodynamic compensation mechanisms fail to respond adequately to the decrease in plasma volume caused by ultrafiltration. This leads to reduced cardiac output, impaired peripheral vasoconstriction and refilling capacity, and decreased MAP [107]. If cerebral autoregulation is impaired, excessive ultrafiltration with decreased MAP might lead to reduced cerebral perfusion and CPP, potentially resulting in decreased ICP.

RRT has the potential to reduce brain tissue oxygenation through multiple pathways. One mechanism involves dialysis-related brain edema, which can impede oxygen diffusion. Additionally, increased ICP associated with a hypermetabolic state in the brain may increase oxygen consumption [108]. Dialysis-induced inflammation could result in pulmonary leukosequestration, leading to reduced arterial oxygen levels. This can impact the brain's respiratory center perfusion and metabolism, ultimately decreasing cerebral oxygenation and causing intermittent short apneic episodes [109]. In a recent study involving 17 adult hemodialysis patients, magnetic resonance imaging, diffusion tensor imaging, and proton magnetic resonance spectroscopy demonstrated that a single hemodialysis session could lead to an increase in brain tissue volume during the session [110]. This change was accompanied by alterations in white matter diffusion metrics and brain metabolite concentrations consistent with ischemic injury [110].

### RRT timing

RRT is generally advised for critically ill patients when absolute solute/volume criteria are met and medical treatment proves insufficient [111]. However, RRT initiation should not be delayed in patients with ABI since rapid changes in osmolality could create an osmotic gradient with adverse neurologic consequences. Despite the association between pre-RRT BUN level and subsequent ICP elevation [112], there is no conclusive evidence on the optimal timing of RRT initiation in patients with ABI. Clinical practice suggests that maintaining pre-dialysis BUN under 30–35 mg/dL with optimized RRT could decrease the risk of increasing ICP during treatment [103].

### RRT modalities

The RRT modalities commonly used in critically ill patients include intermittent hemodialysis (IHD), continuous RRT (CRRT), and prolonged intermittent RRT (PIRRT); (Table 2). IHD is typically used three times weekly for 3–4 h per session and allows for rapid solute control and fluid removal. CRRT encompasses various modalities developed specifically for critically ill patients to provide slower solute and fluid removal than IHD and maintain better hemodynamic stability. PIRRT combines the advantages of both, using conventional hemodialysis machines with blood-pump speeds and dialysate flow rates between IHD and CRRT. PIRRT improves hemodynamic stability through slow solute and fluid removal while avoiding the need for 24-h therapy (Table 3).

**Table 2:** RRT modalities and parameters

Modality	Duration (h)	Frequency	Replacement (mL/min)	Blood (mL/min)	Dialysate (mL/min)	Net ultrafiltration (mL/kg/h)
IHD	3–4	3–4 × /week	–	300–400	600–800	0–10
PIRRT	6–12	3–6 × /week	–	200–300	200–300	0–8
CRRT	24	Daily	None or 16–50	100–300	None or 16–50	0–2

*CRRT* continuous renal replacement therapy, *IHD* intermittent hemodialysis, *PIRRT* prolonged renal replacement therapy, *RRT* renal replacement therapy

**Table 3:** Modifications to intermittent dialytic techniques (IHD and PIRRT) to prevent further ABI

Modification item	Recommendation
Dialyzer	Use membranes with small surface area
blood flow	If using IHD or PIRRT, consider using lower blood flows of < 300 mL/min
Dialysate flow	Consider using lower dialysate flow rates for IHD/PIRRT (< 600 mL/min) and lower effluent rates for CRRT (20 mL/kg/h)
Ultrafiltration Rate	Avoid high net ultrafiltration rates (< 10 mL/kg/h for IHD and < 2 mL/kg/h for CRRT)
Vasopressor	Consider using vasopressors to keep MAP and CPP goals
Dialysate temperature	Cool dialysate to 35 °C
Electrolytes	Use higher dialysate sodium concentration Use lower dialysate bicarbonate concentration Use higher dialysate calcium concentration
Frequency	Daily dialysis to minimize peaks and troughs in serum BUN levels

The table summarizes possible modifications to RRT prescription parameters in patients with ABI [113, 114]

*ABI* acute brain injury, *BUN* blood urea nitrogen, *CPP* cerebral perfusion pressure, *CRRT* continuous renal replacement therapy, *IHD* intermittent hemodialysis, *MAP* mean arterial pressure, *PIRRT* prolonged intermittent renal replacement therapy

CRRT is less likely to cause DDS and intradialytic hypotension than IHD [112, 115] as its slower blood and dialysate/replacement fluid flow rates and smaller dialyzer surface area result in decreased urea clearance from the plasma (Table 3). Brain computed tomography studies showed increased brain water content after IHD but not after CRRT [116]. Moreover, CRRT slower net ultrafiltration rate facilitates hemodynamic stability, prevents intradialytic hypotension, and maintains CPP and brain tissue oxygenation. Although IHD remains an option in patients with mild brain injury and stable conditions, the 2012 KDIGO guidelines recommend CRRT for patients with ABI requiring RRT [6].

There are several strategies to prevent intradialytic hypotension. Common ones include establishing and adjusting the patient’s dry (target) weight; more frequent and longer RRT sessions to be able to decrease ultrafiltration rates; the avoidance of meals during RRT to mitigate the postprandial drop in blood pressure; the preemptive pharmacological use of midodrine, droxidopa, or sertraline; the use of cool dialysate; sodium profiling; and the use of a high calcium bath [117–119].

Conclusive evidence comparing the clinical outcomes after CRRT versus PIRRT in patients with ABI is lacking. A study in patients with brain hemorrhage found similar effects on hemodynamics and ICP [120]. PIRRT is potentially more efficient in resource utilization and offers greater patient care flexibility since it requires a standard IHD device and its administration time is shorter [121]. While the PIRRT effect is intermediate between IHD and CRRT, it could be an alternative to CRRT in patients who might not necessarily require CRRT or with concerns regarding IHD use in ABI and in centers without CRRT availability [68].

### Sodium regulation with RRT

Sodium-based osmotherapy is crucial in managing cerebral edema and preventing ICP increase in patients with ABI. However, the standard sodium concentration in CRRT solutions is 140 mmol/L, lower than the recommended serum sodium concentration to maintain the osmotic pressure between the brain and the plasma (145–155 mmol/L). Sodium concentration in CRRT can be adjusted by adding hypertonic saline (NaCl 23.4%) to the CRRT solution bags or by administering hypertonic saline (NaCl 3%) as CRRT post-filter replacement fluid or as a separate infusion (Table 4). In IHD, a 145 mmol/L sodium bath and a separate hypertonic saline infusion could help achieve the desired serum sodium concentration.

**Table 4:** Pros and cons of adjusting sodium concentration by methods

Method	Pros	Cons	Example calculation
Adding sodium to CRRT solution bags	No extra solutions needed No extra volume added to the patient	Once added, cannot change the sodium concentration of the bag Requires pharmacy services for compounding of solutions	Adding 10 mL/20 mL of 23.4% sodium solution to a 5 L CRRT bag with sodium concentration of 140 mmol/L raises the sodium level to 148/156 mmol/L, respectively
Delivering hypertonic sodium solution through the CRRT machine as post-filter replacement fluid	Volume of the solution accounted by CRRT device Easy to adjust the rate of administering hypertonic sodium solution	Requires a CRRT device and a CRRT modality that allows for post-filter replacement fluid	3% infusion rate = [(target Na – 140 mmol/L)/(513 mmol/L – target Na)] × effluent rate in mL/h
Delivering hypertonic sodium solution as a separate infusion through central venous catheter	Easy to adjust the rate of administering hypertonic sodium solution It can be stopped independently of CRRT at any time	Rapid change in serum sodium concentration may occur (e.g., when CRRT is unexpectedly discontinued) Additional volume of hypertonic sodium solution is administered to the patient	3% infusion rate = [(target Na – 140 mmol/L)/(513 mmol/L – target Na)] × effluent rate in mL/h

Adapted from Yessayan et al. [122]

*BUN* blood urea nitrogen, *CPP* cerebral perfusion pressure, *CRRT* continuous renal replacement therapy, *IHD* intermittent hemodialysis, *MAP* mean arterial pressure, *PIRRT* prolonged intermittent renal replacement therapy

### Anticoagulation for RRT

Systemic anticoagulation for RRT should be avoided in patients with ABI. While most intermittent IHD sessions can be conducted without anticoagulation, regional citrate anticoagulation (RCA) is recommended for CRRT [68]. However, some considerations are important. The use of citrate can lead to either metabolic acidosis or alkalosis, depending on the ability of the liver to metabolize citrate. Additionally, due to its calcium-chelating effect, careful calcium replacement is necessary to prevent neurotoxicity stemming from low ionized calcium levels in the patient. RCA should not be used in cases of shock liver or lactic acidosis exceeding 4 mmol/L due to citrate accumulation. Therefore, careful monitoring of acid–base status, calcium, and lactic acid is recommended. If RCA is unavailable or contraindicated, CRRT can be performed without anticoagulation. Although nafamostat could be an alternative anticoagulant [123–126], its efficacy in multiple CRRT clinical settings, including ABI, requires further evaluation.

## Conclusions

AKI, common in neurocritical patients, is associated with increased morbidity and mortality and has significant implications for managing ABI and its sequelae. Prompt identification of the cause of AKI, with a focus on reversible factors, and the adoption of preventive measures are crucial. The use of RRT in ABI patients is challenging due to potential negative impacts on ICP, CPP, brain oxygenation, and more. CRRT is preferred in ABI cases for gradual solute, electrolyte, and volume adjustments. Unless contraindicated, RCA should be considered for anticoagulation during CRRT. When CRRT is unavailable, intermittent dialysis methods with careful adjustments might be used to minimize complications. Given the magnitude of the problem, future research should focus on better understanding the mechanisms leading to AKI during ABI, and optimizing AKI management in neurocritical care.

### Supplementary Information

**Additional file 1: Appendix Table 1.** Common causes of AKI in the ICU setting.

**Additional file 2.** Appendix Literature Search Strategy.

**Additional file 3: Appendix Table 2.** AKI and RRT rates and associated outcomes in neurocritically ill patients.

**Additional file 4: Appendix Table 3.** Current and proposed definition and staging of AKI.

**Additional file 5: Appendix Table 4.** Characteristics of novel AKI biomarkers.

## Data Availability

Not applicable.

## Abbreviations

ABI: Acute brain injury
AKI: Acute kidney injury
ATACH-II: Antihypertensive treatment of acute cerebral hemorrhage II
BP: Blood pressure
CKD: Chronic kidney disease
COBI: Continuous hyperosmolar therapy for traumatic brain-injured patients
CPP: Cerebral perfusion pressure
CRRT: Continuous renal replacement therapy
DDS: Dialysis disequilibrium syndrome
EPO-TBI: Erythropoetin in traumatic brain injury
ESICM: European Society of Intensive Care Medicine
GFR: Glomerular filtration rate
ICH: Intracerebral hemorrhage
ICP: Intracranial pressure
ICU: Intensive care unit
IHD: Intermittent hemodialysis
KDIGO: Kidney Disease: Improving Global Outcomes
MAP: Mean arterial pressure
NCS: Neurocritical Care Society
PIRRT: Prolonged intermittent renal replacement therapy
RCA: Regional citrate anticoagulation
RRT: Renal replacement therapy
SAH: Subarachnoid hemorrhage
SBP: Systolic blood pressure
SCr: Serum creatinine
TBI: Traumatic brain injury
UOP: Urinary output
