# Supplementary material for: Acute kidney injury in neurocritical care
Source: Crit Care. 2023 Sep 3;27:341. doi: 10.1186/s13054-023-04632-1 (PMC10475203; doi:10.1186/s13054-023-04632-1)
Supplement: Supplementary file 1 — Additional file 1: Appendix Table 1. Common causes of AKI in the ICU setting. [file 13054_2023_4632_MOESM1_ESM.docx]

**Appendix Table 1. Common causes of AKI in the ICU setting**

| **Etiology** | **Definition** | **Epidemiology** | **Pathophysiology** | **Management** |
| --- | --- | --- | --- | --- |
| Sepsis-associated AKI | No consensus definition; the ADQI workgroup proposed the presence of both consensus sepsis criteria (by Sepsis-3 recommendation) and AKI criteria (by KDIGO recommendations) when AKI occurs within 7 days from diagnosis of sepsis [1] | Most common cause of AKI (45%–70% of AKI cases in the ICU){Peerapornratana, 2019 #4734}{Peerapornratana, 2019 #4734}{Peerapornratana, 2019 #4734}{Peerapornratana, 2019 #4734}{Peerapornratana, 2019 #4734}{Peerapornratana, 2019 #4734} | No longer though to be primarily ischemic/hypotensive in nature [1]; key factors are:   - Redistribution of intrarenal perfusion due to altered vascular tone/function and shunting - Inflammation - Oxidative stress - immunological and autonomic dysregulation - abnormal cellular response to injury | Source control, treatment with antimicrobials, fluid resuscitation, and vasopressor medication |
| Cardiac surgery-associated AKI | No consensus definition, but KDIGO criteria are becoming standard | Second most common cause of AKI (20%–25% of AKI cases in the ICU [2]) | Key factors are:   - Renal ischemia–reperfusion injury due to extracorporeal circulation - Hemolysis - Inflammation - Oxidative stress | In the PrevAKI trial [3], early implementation of KDIGO care bundles reduced AKI rates by 10%–15% in a high-risk population |
| Cardiorenal syndrome | - Type 1: Acute cardiovascular disease scenario leading to AKI - Type 3: AKI causing cardiac dysfunction - Type 5: Systemic conditions causing simultaneous dysfunction of the heart and kidney [4] | 45%–65% of patients with heart failure with reduced ejection fraction develop kidney disease [5] | - Type 1: Kidney arterial underfilling and increased venous congestion due to right ventricular dysfunction - Type 3: Incompletely understood; myocardial contractility defects likely due to inflammatory infiltrates in the cardiac tissue - Type 5: Sepsis is the most common example in the ICU | Stepped pharmacologic therapy to relieve congestion; mild- to moderate-sized increments in SCr during decongestive therapy not associated with negative outcomes and rather reflect glomerular hemodynamic changes without tubule cell damage (so-called functional AKI); SGLT2-i therapy improves congestion status in acute decompensated heart failure and is not associated with AKI [6] |
| Hepatorenal syndrome-AKI | - Absolute increase in SCr ≥0.3 mg/dL within 48 h or increase in SCr >1.5 times baseline level; or urine output <0.5 mL/kg/hr for 6 h [7] - Cirrhosis with ascites - No improvement in SCr after 48 h of diuretic withdrawal and volume expansion with albumin - No current or recent use of nephrotoxic drugs - Proteinuria <500 mg/d and hematuria <50 red blood cells per high-power field, or no abnormal renal ultrasonography | 50% of patients with cirrhosis disease develop AKI [8] | Primarily due to intense renal vasoconstriction without structural kidney damage as a result of splanchnic arterial vasodilation and central hypovolemia; may overlap with acute cardiorenal syndrome from cirrhotic cardiomyopathy or right ventricular failure from portopulmonary hypertension | - Albumin (1 g/kg up to a maximum of 100 g daily) - Terlipressin (first-line agent) - Noradrenaline (requires continuous infusion in ICU) - Midodrine + Octreotide (for max 24–48 h, and only if terlipressin not available or contraindicated) - Liver transplant |
| Intra-abdominal hypertension | Intra-abdominal hypertension defined as IAP >12 mm Hg; ACS defined as IAP >20 mm Hg associated with new organ dysfunction | Linear relationship between intra-abdominal hypertension and severity of decreased renal function [9] | Similar to CRS and HRS, key factors are:   - Decreased renal perfusion and increased interstitial pressure (the latter due to increased venous congestion and/or ureteral compression) - Systemic inflammation due to impaired visceral perfusion contribute to renal damage | - Paracentesis of ascites - Correct positive fluid balance (avoidance of excessive fluid resuscitation; use of diuretics) - Adequate sedation and analgesia to control abdominal muscle tone - Decompressive laparotomy |
| Nephrotoxic exposure | Nephrotoxic drugs (e.g., anti-infective drugs [vancomycin, piperacillin/tazobactam, aminoglycosides, acyclovir, amphotericin], iodinated contrast media, hydroxyethyl starch, mannitol) and endogenous toxins (e.g., myoglobin, uric acid) causing decrease in renal function | Depending on the toxin, its concentration on the tubular level, and underlying severity of kidney disease | Key factors involve [10]:   - Direct cytotoxic effects on kidney tubular epithelial or endothelial cells - Impaired kidney hemodynamics - Precipitation of metabolites or crystals, or formation of tubular casts - may cause interstitial nephritis (see below) | - Avoidance and/or limited use of nephrotoxic agents, particularly in patients with kidney disease - Drug level monitoring |
| AKI associated with malignancy | KDIGO-defined AKI | Up to 54% of patients with cancer develop AKI in the ICU setting [11] | Cancer-specific causes may include [11]:   - Nephrotoxic chemotherapy - Cast nephropathy - Lymphomatous infiltration/obstructive nephropathy - Thrombotic microangiopathy - Tumor lysis syndrome - Hypercalcemia - Glomerular diseases | Discontinuation/Reduction of causing agent if possible; treatment of underlying condition |
| Rapidly progressive glomerulonephritis | Rapid decrease in kidney function, with the histologic counterpart of crescentic glomerulonephritis | Rare disease (7 cases/million population per year) [12] | In genetically predisposed patients, different agents may activate autoimmune response resulting in glomerular inflammation and injury (e.g., ANCA-associated vasculitis, anti-glomerular basement membrane disease) | Depending on underlying disease; generally immunosuppressive agents |
| Acute interstitial nephritis | Defined as “acute kidney inflammation characterized by cellular and fluid exudation in the interstitial tissue, accompanied by, but not dependent on, degeneration of the epithelium; the exudation is not purulent in character, and the lesions may be both diffuse and focal” [13] | Incidence ranges from 1%–3% in patients undergoing kidney biopsy for any indication [13] | In genetically predisposed patients, drugs or infectious agents may active autoimmune reaction, resulting in interstitial inflammatory cell infiltration, potentially resulting in interstitial fibrosis | Discontinuation of causing agent if possible (e.g., penicillins, quinolones, NSAIDs, phenytoin, proton pump inhibitors, allopurinol); consider short course of corticosteroids |
| Post-renal AKI | Decrease in kidney function due to extrarenal (e.g., prostate hypertrophy, cancer) or intrarenal (e.g., nephrolithiasis) obstruction; AKI requires bilateral obstruction or unilateral obstruction in a patient with solitary kidney or CKD | ~10% of overall AKI in hospitalized patients [14] | Obstruction leads to an increase in intratubular pressure, impaired renal blood flow, and inflammatory processes resulting in decrease of kidney function, depending on baseline function and the severity of obstruction | - Treatment of the underlying cause - Bladder catheterization - Double-J urethral stent |

ACS, acute compartment syndrome; ADQI, Acute Dialysis Quality Initiative; AKI, acute kidney injury; CKD, chronic kidney disease; CRS; cardiorenal syndrome; HRS, hepatorenal syndrome; IAP, intra-abdominal pressure; ICU, intensive care unit; KDIGO, Kidney Disease: Improving Global Outcomes; NSAID, non-steroidal anti-inflammatory drug; SOFA, Sequential (Sepsis-related) Organ Failure Assessment; SCr, serum creatinine.

**References**

1. Zarbock A, Nadim MK, Pickkers P, Gomez H, Bell S, Joannidis M, Kashani K, Koyner JL, Pannu N, Meersch M, et al. Sepsis-associated acute kidney injury: consensus report of the 28th Acute Disease Quality Initiative workgroup. Nat Rev Nephrol. 2023;19(6):401-417.

2. Hu J, Chen R, Liu S, Yu X, Zou J, Ding X. Global Incidence and Outcomes of Adult Patients With Acute Kidney Injury After Cardiac Surgery: A Systematic Review and Meta-Analysis. J Cardiothorac Vasc Anesth. 2016;30(1):82-89.

3. Zarbock A, Kullmar M, Ostermann M, Lucchese G, Baig K, Cennamo A, Rajani R, McCorkell S, Arndt C, Wulf H, et al. Prevention of Cardiac Surgery-Associated Acute Kidney Injury by Implementing the KDIGO Guidelines in High-Risk Patients Identified by Biomarkers: The PrevAKI-Multicenter Randomized Controlled Trial. Anesth Analg. 2021;133(2):292-302.

4. Rangaswami J, Bhalla V, Blair JEA, Chang TI, Costa S, Lentine KL, Lerma EV, Mezue K, Molitch M, Mullens W, et al. Cardiorenal Syndrome: Classification, Pathophysiology, Diagnosis, and Treatment Strategies: A Scientific Statement From the American Heart Association. Circulation 2019, 139(16):e840-e878.

5. House AA. Management of Heart Failure in Advancing CKD: Core Curriculum 2018. Am J Kidney Dis. 2018;72(2):284-295.

6. Schulze PC, Bogoviku J, Westphal J, Aftanski P, Haertel F, Grund S, von Haehling S, Schumacher U, Mobius-Winkler S, Busch M. Effects of Early Empagliflozin Initiation on Diuresis and Kidney Function in Patients With Acute Decompensated Heart Failure (EMPAG-HF). Circulation. 2022;146(4):289-298.

7. Angeli P, Garcia-Tsao G, Nadim MK, Parikh CR. News in pathophysiology, definition and classification of hepatorenal syndrome: A step beyond the International Club of Ascites (ICA) consensus document. J Hepatol. 2019;71(4):811-822.

8. Nadim MK, Garcia-Tsao G. Acute Kidney Injury in Patients with Cirrhosis. New Engl J Med. 2023;388(8):733-745.

9. De Waele JJ, De Laet I, Kirkpatrick AW, Hoste E. Intra-abdominal Hypertension and Abdominal Compartment Syndrome. Am J Kidney Dis. 2011;57(1):159-169.

10. Ronco C, Bellomo R, Kellum JA. Acute kidney injury. Lancet. 2019;394(10212):1949-1964.

11. Rosner MH, Perazella MA. Acute Kidney Injury in Patients with Cancer. New Engl J Med. 2017;376(18):1770-1781.

12. Saha MK, Pendergraft III WF, Jennette JC, Falk RJ. Primary Glomerular Disease. In: Yu ASL, Chertow GM, Luyckx VA, Mardsen PA, Skorecki K, Taal MW: Brenner & Rector’s The Kidney. United States: Elsevier; 2020:1072.

13. Perazella MA RMTDIYA, Chertow GM, Luyckx VA, Mardsen PA, Skorecki K, Taal MW: Brenner & Rector’s The Kidney. United States: Elsevier; 2020:1204.

14. Judd E, Sanders PW, Agarwal A. Diagnosis and Clinical Evaluation of Acute Kidney Injury. In: Feehally J, Floege J, Tonelli M, Johnson RJ: Comprehensive Clinical Nephrology. United States: Elsevier; 2019:810.
