# Supplementary material for: Acute kidney injury in neurocritical care
Source: Crit Care. 2023 Sep 3;27:341. doi: 10.1186/s13054-023-04632-1 (PMC10475203; doi:10.1186/s13054-023-04632-1)
Supplement: Supplementary file 2 — Additional file 2. Appendix Literature Search Strategy. [file 13054_2023_4632_MOESM2_ESM.docx]

**Appendix Literature Search Strategy**

We searched PubMed from database inception to July 10, 2023, using the terms "neurocritical" AND "acute kidney injury" AND/OR "tubular biomarker", "neuro intensive care" AND "acute kidney injury" AND/OR "tubular biomarker", "neurocritical" AND "renal recovery", "neuro intensive care" AND " renal recovery", "neurocritical" AND "sepsis" AND "acute kidney injury", "neurocritical" AND "rhabdomyolysis", "brain renal crosstalk”, brain kidney crosstalk”, "neurocritical" AND "fluid balance", "traumatic brain injury" AND "acute kidney injury", "neurogenic stunned myocardium" AND "acute kidney injury", "acute stroke" AND "acute kidney injury", "intracerebral hemorrhage" AND "acute kidney injury", "subarachnoid hemorrhage" AND "acute kidney injury", "neurocritical" AND "augmented renal clearance", "neuro intensive care" AND "augmented renal clearance". No additional filters were applied. The titles and abstracts of the articles resulting from these searches were screened manually. Only English and German language manuscripts were selected. All randomized controlled trials were read in full, and their reference lists were reviewed. Prospective and retrospective studies were analyzed by the abstract and, if required, read in full. To allow comparison of the epidemiology of AKI across studies, we only selected studies that used RIFLE (Risk, Injury, and Failure, sustained Loss and End-stage renal disease), AKIN (Acute Kidney Injury Network), or KDIGO (Kidney Disease: Improving Global Outcomes) criteria for acute kidney injury diagnosis and staging. Only studies involving adult population were considered. Relevant guidelines and clinical practice recommendations relevant to the field were also reviewed.
