# Supplementary material for: Acute kidney injury in neurocritical care
Source: Crit Care. 2023 Sep 3;27:341. doi: 10.1186/s13054-023-04632-1 (PMC10475203; doi:10.1186/s13054-023-04632-1)
Supplement: Supplementary file 3 — Additional file 3: Appendix Table 2. AKI and RRT rates and associated outcomes in neurocritically ill patients. [file 13054_2023_4632_MOESM3_ESM.docx]

**Appendix Table 2. AKI and RRT rates and associated outcomes in neurocritically ill patients**

| **Study** | **Design** | **Population** | **Exclusion criteria** | **AKI definition** | **AKI rate** | **RRT rate** | **Outcomes** |
| --- | --- | --- | --- | --- | --- | --- | --- |
| **Traumatic brain injury** | | | | | | | |
| Li et al. (2011) [1] | Retrospective single-center (*n* = 136) | Age ≥16 years with severe TBI (GCS ≤ 8) admitted to neurosurgical ICU | Pre-existing kidney disease (not defined); ICU stay <48 h | SCr and UOP (AKIN) | Overall, 23% (stage 1: 15.7%; stage 2: 5.1%; stage 3: 2.3%) | – | 94% developed AKI within a week after TBI; mortality rate in AKI higher than in non-AKI (17% vs. 11%; *P*<0.001) |
| Maguigan et al. (2017) [2] | Retrospective single-center (*n* = 162) | Adults with severe TBI (GCS <9) with ≥2 doses of hypertonic sodium solution admitted to trauma unit | Craniotomy due to elevated ICP | SCr (RIFLE) | 10.5% | – | AKI more common in continuous than bolus infusion group (12.9% vs. 0%; *P*=0.025) |
| Luu et al. (2021) [3] | U.S. registry study using the National Trauma Data Bank (*n* = 37,851) | Adults with severe TBI (GCS ≤8) | CKD (not defined); ICU stay <48 h | SCr (AKIN) | 2.1% (stage 3) | – | Severe AKI associated with in-hospital mortality (OR, 2.03; 95% CI, 1.64–2.52), need for tracheostomy (OR, 2.10; 95% CI, 1.52–2.89), and PEG tube placement (OR, 1.88; 95% CI, 1.45–2.45) |
| Robba et al. (2021) [4] | Prospective multicenter (*n* = 1,262) | Adults with TBI | Presented >24 h of injury; ICU stay ≤72 h; <2 SCr values during first 7 days of ICU stay | SCr (KDIGO) | Overall, 11.9% (stage 1: 7.8%; stage 2: 1.1%; stage 3: 2.9%) | Overall, 1.8% | AKI occurrence within a median of 2 (IQR, 1–4) days; patients with AKI monitored with ICP more frequently than no-AKI (71.9% vs. 61.1%, *P*=0.019), and associated with higher max. Na value (151±9 vs. 147±6 mmol/L) and respiratory failure rate (42.7% vs. 31.0%; *P*=0.006); CKD, diabetes, osmotic therapy, and Na values ≥150 mmol/L in first 3 days post-admission associated with AKI |
| Skrifvars et al. (2021) [5] | Prospective multicenter (*n* = 568) | Age ≥15 years with moderate-to-severe TBI (GCS ≤12) | Multiple in the context of a randomized trial; a.o. GCS = 3 and fixed pupils; CKD G5D | SCr (KDIGO) | 14.0%, using admission SCr as baseline | – | AKI occurrence within a median of 6 (IQR, 3–8) days; mannitol on day 1 or 2, but not hypertonic sodium solution, associated with increased risk of AKI (OR, 2.9; 95% CI, 1.3–6.7) |
| Wang et al. (2021) [6] | Retrospective single-center (*n* = 419) | Patients with severe TBI (GCS ≤9) admitted to neurosurgery | CKD (not defined); admission ≥6 h after injury | SCr (KDIGO) | Overall, 19.8% (stage 1: 9.7%; stage 2: 3.4%; stage 3: 6.7%) | – | AKI occurrence within a median of 1 (IQR, 1–2) days; AKI occurrence (OR, 3.79; *P*=0.004) and highest AKI stage (OR, 3.12; *P*<0.01) associated with mortality |
| Huang et al. (2022) [7] | Retrospective single-center (*n* = 1,648) | Patients with severe TBI (GCS ≤9) admitted to NICU | CKD G5D; ICU stay ≤24 h | SCr and UOP (RIFLE, AKIN, KDIGO) | KDIGO: 17.7%; AKIN: 17.1%; RIFLE: 12.7% | – | KDIGO criteria detect highest proportion of AKI patients |
| Liu et al. (2023) [8] | Retrospective single-center (*n* = 2,470) | Adults with TBI admitted to ICU (mild: 48.5%, moderate: 13.7%, severe: 37.9%) | AKI within 24 h of admission | SCr (KDIGO) | Overall, 21.0% (stage 1: 16.7%; stage 2; 3.2%; stage 3: 1.2%) | – | Serum magnesium level <1.5 mg/dL independently associated with AKI |
| Huet et al. (2023) [9] | Prospective, multicenter, randomized trial (post-hoc analysis of the COBI trial; *n* = 322) | Adults with moderate-to-severe TBI (GCS ≤12) randomized to continuous hypertonic sodium solution for at least 48 h (goal sodium level 150–155 mmol/L) | Time from symptoms to ICU randomization >24 h; GCS = 3; fluid retention | SCr (KDIGO) | 26.7% | Overall, 1.9% | Similar AKI rates in the intervention and control group (24.5% vs. 28.9%; *P*=0.45) |
| **Aneurysmal and non-aneurysmal subarachnoid hemorrhage** | | | | | | | |
| Zacharia et al. (2009) [10] | Prospective single-center (*n* = 787) | Adults with aneurysmal SAH admitted to NICU | – | SCr (RIFLE) | 23.1% (Risk stage) | – | AKI associated with poor 3-months survival and functional outcome (OR, 2.01; *P*<0.021) |
| Kumar et al. (2014) [11] | Retrospective single-center (*n* = 736) | Adults with aneurysmal and non-aneurysmal SAH admitted to NICU | CKD G5D; ICU stay <24 h | SCr (AKIN) over 14 days | 9.0% | Overall, 0.3% | AKI associated with increased mortality (OR, 2.33; 95% CI, 1.27–4.30); hypertonic solution strongest risk factor for AKI; for each 1 mmol/L increase in serum Na, AKI hazard increases by 5.4% (95% CI, 1.4–9.7) |
| Sadan et al. (2017) [12] | Retrospective single-center (*n* = 1,267) | Adults with aneurysmal SAH admitted to NICU | ICU stay <6 h; readmission | SCr (KDIGO) | 16.7% | – | Mortality rate higher in AKI than non-AKI group (28.3% vs. 6.1%; *P*<0.001); renal recovery (discharge minus admission SCr ≤0.3 mg/dL) in 78.8% of AKI cases; mortality in recovered lower than in non-recovered (18.6% vs. 64.4%; *P*<0.001) |
| Tujjar et al. (2017) [13] | Retrospective single-center (*n* = 647) | Adults with aneurysmal SAH admitted to general ICU | Time from symptoms to ICU admission >96 h; ICU stay <48 h; CKD G5D | SCr and UOP (AKIN) | 12.4% | Overall, 0.5% | Median time to AKI: 8 (IQR, 4–10) days; AKI associated with more severe neurological impairment and more aggressive ICU therapy; multivariable regression analysis found no association between AKI and ICU mortality |
| Sadan et al. (2020) [14] | Prospective, single-center, randomized pilot trial (*n* = 59) | Adults with aneurysmal SAH admitted to NICU | CKD G5D | SCr (KDIGO) | 23.7% | – | Median time to AKI: 3 (IQR, 2–7) days; AKI higher in NaCl than NaCl/Na-acetate group (53.3% vs. 11.8%; *P*=0.01); no difference in novel AKI biomarkers |
| **Intracerebral hemorrhage** | | | | | | | |
| Riha et al. (2017) [15] | Retrospective multicenter (*n* = 243; unmatched cohort, *n* = 243; propensity-matched cohort: *n* = 100) | Aged >17 years with ICH receiving continuous IV 3% hypertonic sodium solution admitted to NICU | CKD G5 and G5D | SCr (AKIN) | 26.0% in the unmatched cohort, 20.0% in the propensity-matched cohort | – | AKI more common in hyperchloremic than non-hyperchloremic patients (unmatched cohort 35.0% vs. 9.2%; propensity-matched cohort: 30.0% vs. 10.0%) |
| Burgess et al. (2018) [16] | Prospective single-center (*n* = 448) | Adults with spontaneous ICH admitted to ICU | CKD G5D | SCr (AKIN) | Overall, 29.9% (stage 1: 25.4%; stage 2: 2.9%; stage 3: 1.6%) | – | In first 12 h, SBP reduction >90 mm Hg increases risk of AKI in patients with normal renal function (OR, 2.1; 95% CI, 1.19–3.62) and CKD (OR, 3.91; 95% CI, 1.26–12.15) |
| **Acute stroke** | | | | | | | |
| Lin et al. (2015) [17] | Retrospective single-center (*n* = 432) | Aged >20 years with acute stroke and mannitol infusion admitted to NICU (62.3% with ischemic stroke) | AKI prior to mannitol therapy; CKD G5D | SCr (AKIN) | 6.5% | – | Diabetes, lower baseline eGFR, higher initial NIHSS score, and diuretic use associated with AKI |
| Wang et al. (2018) [18] | Retrospective single-center (*n* = 647) | Adults with stroke admitted to NICU (ischemic stroke: 28.1%, hemorrhagic stroke: 71.9%) | CKD G5 and G5D | SCr (KDIGO) | Overall, 20.9% (stage 1: 13.0%; stage 2: 4.0%; stage 3: 3.9%) | – | Independent risk factors for AKI: higher NIHSS score (OR, 1.03; 95% CI, 1.00–1.05), hypertension (OR, 1.59; 95% CI, 1.00–2.53), and infectious complications (OR, 3.39; 95% CI, 2.00–5.80); AKI severity associated with increased hospital mortality |
| Gwak et al. (2021) [19] | Retrospective single-center (*n* = 90) | Adults with large hemispheric infarction (NIHSS score ≥13) admitted to NICU | CKD G5; ICU stay ≤72 h | SCr (KDIGO) | Overall, 43.3% (stage 1: 15.6%; stage 2; 12.2%; stage 3: 15.6%) | Overall, 5.6% | Maximum serum chloride level associated with AKI occurrence (adjusted OR, 1.57; 95% CI, 1.18–2.08) |
| **Mixed ICU cohort** | | | | | | | |
| Hoste et al. (2015) [20] | Prospective multicenter [*n* = 1,802; neurologic: *n* = 466 (25.9%) / neurosurgery: *n* = 83 (4.6%)] | Adults admitted to ICUs (no report on neurologic conditions) | CKD G5D; kidney transplant recipients; anticipated alive ICU discharge within 24 h | SCr and UOP (KDIGO) | Overall, 57.3% (stage 1: 18.4%; stage 2: 8.9%; stage 3: 30.0%) | Overall, 13.5% | AKI severity associated with increased hospital mortality in the overall cohort (no report on neurologic cohort; stage 1: OR, 2.19; 95 % CI, 1.44–3.35; stage 2: OR, 3.88; 95% CI, 2.42–6.21; stage 3: OR, 7.19; 95% CI, 5.13–10.04; mortality similar across continents and regions |
| Erdman et al. (2017) [21] | Retrospective multicenter (*n* = 329) | Adults with hypertonic sodium solution admitted to NICU (ischemic stroke: 34.4%, ICH: 35.3%, TBI: 11.3%) | CKD G5D | SCr (AKIN) | Overall, 16.4% (stage 1: 8.3%; stage 2: 4.0%; stage 3: 4.0%) | Overall, 0.9% | AKI had longer ICU stay than no AKI (14.8 vs. 11.5 days; *P*=0.006) and higher mortality (48.1% vs. 21.9%, *P*<0.001); serum sodium >155 mmol/L (OR, 4.1; 95% CI, 2.1–8.0) associated with AKI |
| Büttner et al. (2020) [22] | Retrospective single-center (*n* = 681) | Adults admitted to mixed neurosurgical/  neurological ICU (intracranial hemorrhage: 48.5%; CNS neoplasms: 14.5%; ischemic stroke: 14.2%; CNS infection: 11.0%) | ICU stay <48 h | SCr (AKIN) | 11.6% (stage ≥2) | Overall, 5.3% | Sepsis considered as main cause of AKI in 53.2% of AKI patients; AKI had higher hospital mortality than no AKI (38.0% vs. 13.0%; *P*<0.01); AKI (HR, 3.70; 95% CI, 1.87–7.35) and need for RRT (HR, 2.85; 95% CI, 1.30–6.33) independent risk factors for death |
| Sigmon et al. (2020) [23] | Retrospective single-center (*n* = 142) | Adults receiving continuous IV hypertonic sodium solution admitted to NICU (ICH: 28.2%, aneurysmal hemorrhage: 26.1%, ischemic stroke: 26.1%, brain lesion/tumor: 12.7%, others: 6.3%) | CKD G5D | SCr (AKIN) | 12.7% | – | Hyperchloremia rate in AKI higher than in no-AKI (67% vs. 34%; *P*<0.01); patients with >2,055 mmol chloride over 7 days more likely to develop AKI (AUC = 0.70; sensitivity 72%, specificity 70%) |
| Hamilton et al. (2021) [24] | Retrospective single-center (*n* = 133) | Adults admitted to NICU (ischemic stroke: 38.4%, ICH: 33.8%, SAH: 12.0%; TBI: 0.8%; others: 15.0%) | CKD G5 and G5D | SCr and UOP (KDIGO) | 29.3% | – | Serum chloride >113.5 mmol/L associated with AKI occurrence (OR, 3.14; 95% CI, 1.10–9.04) |
| Ramirez-Guerrero et al. (2023) [25] | Retrospective single-center (*n* = 231) | Adults admitted to ICU (SAH: 40.4%; TBI: 38.9%, ICH: 11.7%, stroke: 7.5%; others: 1.4%) | AKI at admission; CKD (eGFR <60 mL/min/1.73 m^2^); CKD G5D; ICU stay ≤48 h | SCr and UOP (KDIGO) over 14 days after admission | Overall, 23.5% (stage 1: 13.6%; stage 2; 6.1%; stage 3: 3.8%) | Overall, 2.0% | Mortality rate in AKI higher than in non-AKI (40.0% vs. 14.5%; *P*<0.001); change in serum chloride >4 mmol/L in 48 h of admission (OR, 2.41; 95% CI, 1.10–5.37) and cerebral edema (OR, 4.40; 95% CI, 1.98–9.75) associated with AKI occurrence |

AKI, acute kidney injury; AKIN, Acute Kidney Injury Network; CKD, chronic kidney disease; CKD G5D; chronic kidney disease stage 5 treated with maintenance dialysis; CNS, central nervous system; eGFR, estimated glomerular filtration rate; ICH, intracerebral hemorrhage; ICU, intensive care unit; IV, intravenous; KDIGO, Kidney Disease: Improving Global Outcomes; NICU, neurological intensive care unit; NIHSS, National Institutes of Health Stroke Scale; PEG, percutaneous endoscopic gastrostomy; RIFLE, Risk, Injury, Failure, Loss, End-stage renal disease; RRT, renal replacement therapy; SAH, subarachnoid hemorrhage; SCr, serum creatinine; TBI, traumatic brain injury; GCS, Glasgow Coma Scale; UOP, urine output.

**References**

1. Li N, Zhao WG, Zhang WF. Acute kidney injury in patients with severe traumatic brain injury: implementation of the acute kidney injury network stage system. Neurocrit Care. 2011;14(3):377-381.

2. Maguigan KL, Dennis BM, Hamblin SE, Guillamondegui OD: Method of Hypertonic Saline Administration. Effects on Osmolality in Traumatic Brain Injury Patients. J Clin Neurosci. 2017;39:147-150.

3. Luu D, Komisarow J, Mills BM, Vavilala MS, Laskowitz DT, Mathew J, James ML, Hernandez A, Sampson J, Fuller M, et al. Association of Severe Acute Kidney Injury with Mortality and Healthcare Utilization Following Isolated Traumatic Brain Injury. Neurocrit Care. 2021;35(2):434-440.

4. Robba C, Banzato E, Rebora P, Iaquaniello C, Huang CY, Wiegers EJA, Meyfroidt G, Citerio G, Collaborative European NeuroTrauma Effectiveness Research in Traumatic Brain Injury ICUP, Investigators. Acute Kidney Injury in Traumatic Brain Injury Patients: Results From the Collaborative European NeuroTrauma Effectiveness Research in Traumatic Brain Injury Study. Crit Care Med. 2021;49(1):112-126.

5. Skrifvars MB, Bailey M, Moore E, Martensson J, French C, Presneill J, Nichol A, Little L, Duranteau J, Huet O, et al. A Post Hoc Analysis of Osmotherapy Use in the Erythropoietin in Traumatic Brain Injury Study-Associations With Acute Kidney Injury and Mortality. Crit Care Med. 2021;49(4):e394-e403.

6. Wang R, Zhang J, Xu J, He M, Xu J. Incidence and Burden of Acute Kidney Injury among Traumatic Brain-Injury Patients. Risk Manag Healthc Policy. 2021;14:4571-4580.

7. Huang ZY, Liu Y, Huang HF, Huang SH, Wang JX, Tian JF, Zeng WX, Lv RG, Jiang S, Gao JL. et al. Acute kidney injury in traumatic brain injury intensive care unit patients. World J Clin Cases. 2022;10(9):2751-2763.

8. Liu Z, Wang R, He M, Kang Y. Hypomagnesemia Is Associated with the Acute Kidney Injury in Traumatic Brain Injury Patients: A Pilot Study. Brain Sci. 2023;13(4).

9. Huet O, Chapalain X, Vermeersch V, Moyer JD, Lasocki S, Cohen B, Dahyot-Fizelier C, Chalard K, Seguin P, Hourmant Y, et al. Impact of continuous hypertonic (NaCl 20%) saline solution on renal outcomes after traumatic brain injury (TBI): a post hoc analysis of the COBI trial. Crit Care. 2023;27(1):42.

10. Zacharia BE, Ducruet AF, Hickman ZL, Grobelny BT, Fernandez L, Schmidt JM, Narula R, Ko LN, Cohen ME, Mayer SA, et al. Renal dysfunction as an independent predictor of outcome after aneurysmal subarachnoid hemorrhage: a single-center cohort study. Stroke. 2009;40(7):2375-2381.

11. Kumar AB, Shi Y, Shotwell MS, Richards J, Ehrenfeld JM. Hypernatremia is a significant risk factor for acute kidney injury after subarachnoid hemorrhage: a retrospective analysis. Neurocrit Care. 2015;22(2):184-191.

12. Sadan O, Singbartl K, Kandiah PA, Martin KS, Samuels OB. Hyperchloremia Is Associated With Acute Kidney Injury in Patients With Subarachnoid Hemorrhage. Crit Care Med. 2017;45(8):1382-1388.

13. Tujjar O, Belloni I, Hougardy JM, Scolletta S, Vincent JL, Creteur J, Taccone FS. Acute Kidney Injury After Subarachnoid Hemorrhage. J Neurosurg Anesthesiol. 2017;29(2):140-149.

14. Sadan O, Singbartl K, Kraft J, Plancher JM, Greven ACM, Kandiah P, Pimentel C, Hall CL, Papangelou A, Asbury WH, et al. Low-chloride- versus high-chloride-containing hypertonic solution for the treatment of subarachnoid hemorrhage-related complications: The ACETatE (A low ChloriE hyperTonic solution for brain Edema) randomized trial. J Intensive Care. 2020:8:32.

15. Riha HM, Erdman MJ, Vandigo JE, Kimmons LA, Goyal N, Davidson KE, Pandhi A, Jones GM. Impact of Moderate Hyperchloremia on Clinical Outcomes in Intracerebral Hemorrhage Patients Treated With Continuous Infusion Hypertonic Saline: A Pilot Study. Crit Care Med. 2017;45(9):e947-e953.

16. Burgess LG, Goyal N, Jones GM, Khorchid Y, Kerro A, Chapple K, Tsivgoulis G, Alexandrov AV, Chang JJ. Evaluation of Acute Kidney Injury and Mortality After Intensive Blood Pressure Control in Patients With Intracerebral Hemorrhage. J Am Heart Assoc. 2018;7(8):e008439.

17. Lin SY, Tang SC, Tsai LK, Yeh SJ, Shen LJ, Wu FL, Jeng JS. Incidence and Risk Factors for Acute Kidney Injury Following Mannitol Infusion in Patients With Acute Stroke: A Retrospective Cohort Study. Medicine (Baltimore). 2015;94(47):e2032.

18. Wang D, Guo Y, Zhang Y, Li Z, Li A, Luo Y. Epidemiology of acute kidney injury in patients with stroke: a retrospective analysis from the neurology ICU. Intern Emerg Med. 2018;13(1):17-25.

19. Gwak DS, Chung I, Kim BK, Lee S, Jeong HG, Kim YS, Chae H, Park CY, Han MK: High Chloride Burden and Clinical Outcomes in Critically Ill Patients With Large Hemispheric Infarction. Front Neurol. 2021;12:604686.

20. Hoste EA, Bagshaw SM, Bellomo R, Cely CM, Colman R, Cruz DN, Edipidis K, Forni LG, Gomersall CD, Govil D, et al. Epidemiology of acute kidney injury in critically ill patients: the multinational AKI-EPI study. Intensive Care Med. 2015;41(8):1411-1423.

21. Erdman MJ, Riha H, Bode L, Chang JJ, Jones GM. Predictors of Acute Kidney Injury in Neurocritical Care Patients Receiving Continuous Hypertonic Saline. Neurohospitalist. 2017;7(1):9-14.

22. Buttner S, Stadler A, Mayer C, Patyna S, Betz C, Senft C, Geiger H, Jung O, Finkelmeier F. Incidence, Risk Factors, and Outcome of Acute Kidney Injury in Neurocritical Care. Journal of intensive Care Med. 2020;35(4):338-346.

23. Sigmon J, May CC, Bryant A, Humanez J, Singh V. Assessment of Acute Kidney Injury in Neurologically Injured Patients Receiving Hypertonic Sodium Chloride: Does Chloride Load Matter? Ann Pharmacother. 2020;54(6):541-546.

24. Hamilton LA, Behal ML, Carter AR, Rowe AS. Patient-Specific Risk Factors Associated With the Development of Hyperchloremia in a Neurocritical Care Intensive Care Unit. J Pharm Pract. 2023;36(1):110-116.

25. Ramirez-Guerrero G, Lucero C, Villagran-Cortes F, Hauway E, Torres-Cifuentes V, Baghetti-Hernandez R, Vera-Calzaretta A, Ronco C, Garay O. Acute kidney injury in neurocritical patients: a retrospective cohort study. International urology and nephrology 2023;55(7):1875-1883.
