# Supplementary material for: Acute kidney injury in neurocritical care
Source: Crit Care. 2023 Sep 3;27:341. doi: 10.1186/s13054-023-04632-1 (PMC10475203; doi:10.1186/s13054-023-04632-1)
Supplement: Supplementary file 4 — Additional file 4: Appendix Table 3. Current and proposed definition and staging of AKI. [file 13054_2023_4632_MOESM4_ESM.docx]

**Appendix Table 3. Current and proposed definition and staging of AKI**

| **Current definition by KDIGO [1]** | | **Proposed definition by ADQI [2]** | | |
| --- | --- | --- | --- | --- |
| **Stage** | **Criteria** | **Stage** | **Functional criteria** | **Damage criteria (biomarker)** |
| **0** | No increase in serum creatinine by >0.3 mg/dL for <48 h or >150% for <7 days and/or urine output not <0.5 mL/kg/h for >6 h | **1S** | See left panel | + |
| **1** | Serum creatinine increased by >0.3 mg/dL for <48 h or >150% for <7 days and/or urine output <0.5 mL/kg/h for >6 h | **1A** | See left panel | - |
|  |  | **1B** | See left panel | + |
| **2** | Serum creatinine increased by >200% and/or urine output <0.5 mL/kg/h for >12 h | **2A** | See left panel | - |
|  |  | **2B** | See left panel | + |
| **3** | Serum creatinine increased by >300% (or >4 mg/dL) and/or urine output <0.3 mL/kg/h for >24 h or anuria >12 h and/or initiation of RRT | **3A** | See left panel | - |
|  |  | **3B** | See left panel | + |

Adapted from Ostermann et al. [2]. The 2012 KDIGO workgroup proposed a consensus definition and staging system of AKI that is based on an increase of serum creatinine and/or the presence of oliguria, both surrogate markers of kidney dysfunction. A combination of damage and functional markers, along with clinical information, might be used for identifying high-risk patients, improving AKI diagnostic accuracy and processes of care, and assisting AKI management. It should be noted that biomarker positivity should be based on its mechanistic relevance to the pathobiology of AKI and defined thresholds, which still require validation.

To convert serum creatinine to mmol/L, multiply by 88.4.

ADQI, Acute Dialysis Quality Initiative; AKI, acute kidney injury; KDIGO, Kidney Disease: Improving Global Outcomes; RRT, renal replacement therapy.

**References**

1. Kidney Disease: Improving Global Outcomes (KDIGO) Acute Kidney Injury Work Group. KDIGO Clinical Practice Guideline for Acute Kidney Injury. Kidney Int Suppl. 2012;(2):1–138.

2. Ostermann M, Zarbock A, Goldstein S, Kashani K, Macedo E, Murugan R, Bell M, Forni L, Guzzi L, Joannidis M, et al. Recommendations on Acute Kidney Injury Biomarkers From the Acute Disease Quality Initiative Consensus Conference: A Consensus Statement. JAMA Netw Open. 2020;3(10):e2019209.
