# Supplementary material for: Acute kidney injury in neurocritical care
Source: Crit Care. 2023 Sep 3;27:341. doi: 10.1186/s13054-023-04632-1 (PMC10475203; doi:10.1186/s13054-023-04632-1)
Supplement: Supplementary file 5 — Additional file 5: Appendix Table 4. Characteristics of novel AKI biomarkers. [file 13054_2023_4632_MOESM5_ESM.docx]

**Appendix Table 4. Characteristics of novel AKI biomarkers**

| **Biomarker** | **Type** | **Sample** | **Function** | **Clinical utility** |
| --- | --- | --- | --- | --- |
| **[TIMP-2]•[IGFBP7]** | Stress | Urine | 21- and 25-kDa proteins, respectively, implicated in G1 cell cycle arrest, which is thought to be a protective mechanism | Increases immediately after cardiac surgery, and peaks at 4–24 h [1]; FDA-approved and CE-marked for clinical use |
| **KIM-1** | Damage | Urine | 39-kDa transmembrane glycoprotein produced by proximal tubular cells; it is released in response to tubular damage and functions in clearing apoptotic cells, and has anti-inflammatory properties | Increases 12–24 h after tubular injury, peaking at 2–3 days [1]; unreliable in the setting of severe inflammation; FDA-approved and CE-marked for preclinical drug development |
| **L-FABP** | Damage | Urine | 14-kDa intracellular lipid chaperone; secreted by proximal tubular epithelial cells during ischemic and hypoxic injury | Specific times from injury to detection of L-FABP unknown; Japanese Ministry of Health, Labour and Welfare-approved for clinical use (early diagnostic of kidney disease or predicting kidney prognosis) |
| **NGAL** | Damage | Urine or plasma | 25-kDa protein that binds to iron–siderophore complexes that act as chelators/transporters in several diseases | Peaks at 4–6 h after tubular injury [1]; systemic levels are elevated in sepsis and severe inflammation, thus clinical use is limited in the ICU setting; CE-marked for diagnostic use in Europe since 2009 but not FDA-approved for clinical use in the USA |
| **Cystatin C** | Dysfunction | Plasma | 13.3-kDa protein produced in all nucleated cells throughout the body; its function is unknown | Freely filtered at the glomerulus, reabsorbed, and catabolized by the proximal tubule cells, thus plasma levels are indicative of GFR [2]*; FDA-approved and CE-marked for clinical use |
| **Proenkephalin A** | Dysfunction | Plasma | 4.5-kDa protein derived from the precursor enkephalin, a small endogenous opioid peptide produced in the central nervous system, heart, kidney, intestine, lung, skeletal muscle, and immune cells | Freely filtered at the glomerulus, thus plasma levels are indicative of GFR [1]*; CE-marked for clinical use but not FDA-approved for clinical use in the USA |

*Filtration markers have a variable relationship to injury, thus specific times from injury to detection of the markers are not possible to establish.

Appendix Table 4 only summarizes few of the currently available AKI biomarkers. Research is needed to establish which biomarkers will be qualified for augmenting AKI classification. Furthermore, it remains unclear when these biomarkers should be measured and what the cutoffs should be; further investigation is needed.

CE, Conformité Européenne; FDA, US Food and Drug Administration; GFR; glomerular filtration rate; IGFBP7, insulin-like growth factor binding protein; KIM-1, kidney injury molecule 1; L-FABP, liver-type fatty acid-binding protein; NGAL, neutrophil gelatinase-associated lipocalin; TIMP-2, tissue inhibitor of metalloproteinases-2.

**References**

1. Ronco C, Bellomo R, Kellum JA. Acute kidney injury. Lancet. 2019;394(10212):1949-1964.

2. Levey AS, Coresh J, Tighiouart H, Greene T, Inker LA. Measured and estimated glomerular filtration rate: current status and future directions. Nat Rev Nephrol. 2020;16(1):51-64.
